# Relationship between antifungal susceptibility profile and virulence factors in *Candida albicans* isolated from nail specimens

**DOI:** 10.1590/0037-8682-0214-2019

**Published:** 2020-02-07

**Authors:** Faezeh Mohammadi, Zeinab Ghasemi, Behnaz Familsatarian, Eelham Salehi, Somayeh Sharifynia, Ameneh Barikani, Monirsadat mirzadeh, Mohammad Ali Hosseini

**Affiliations:** 1 Medical Microbiology Research Center, Qazvin university of Medical Science, Qazvin, Iran.; 2 Medical Mycology of Razi Hospital, Tehran, Iran.; 3 Cellular and Molecular Research Center, Qazvin University of Medical Sciences, Qazvin, Iran.; 4 Clinical Tuberculosis and Epidemiology Research Center, Shahid Beheshti University of Medical Sciences, Tehran, Iran.; 5 Children Growth Research Center, Qazvin University of Medical Science, Qazvin, Iran.; 6 Metabolic Disease Research Center, Qazvin University of Medical Sciences, Qazvin, Iran.; 7 Student Research Committee, Qazvin University of Medical Sciences, Qazvin, Iran.

**Keywords:** Candida albicans, Virulence factors, Biofilm, Antifungal agents

## Abstract

**INTRODUCTION:**

The aim of this study was to evaluate some virulence factors in *Candida albicans* isolates from patients with onychomycosis and determine the correlation between these factors and the antifungal resistance profile.

**METHODS:**

Seventy species of *C. albicans* were confirmed using polymerase chain reaction amplification of the *HWP1* gene. According to the Clinical & Laboratory Standards Institute guidelines, the susceptibility profile of four antifungal agents was investigated, and the production of aspartyl protease, phospholipase, haemolysin, and biofilm was determined. The correlation between these profiles was also investigated.

**RESULTS:**

The isolates indicated different levels of resistance and production of virulence factors. Significant correlations were observed between the minimum inhibitory concentration (MIC) of fluconazole/itraconazole and biofilm production, between phospholipase production and fluconazole/itraconazole MIC, and between fluconazole MIC and hemolytic activity in *C. albicans* isolates*.* The results also showed significant correlations between phospholipase activity and biofilm production.

**CONCLUSIONS:**

Our findings will contribute to a better understanding of the pathogenesis of *C. albicans* and characterize the relationship between virulence factors and antifungal resistance, which may suggest new therapeutic strategies considering the possible involvement of the virulence mechanism in the effectiveness of treatment.

## INTRODUCTION

*Candida* species are opportunistic fungi that are involved in a wide range of superficial to systemic diseases. Among non-invasive infections, onychomycosis, which is a fungal nail infection caused by *Candida* species*,* is the most common cause of onychomycosis following dermatophytes^1^ ^,^ ^2^. Due to the increase in the number of patients with immunodeficiency, as well as changes in fungal pathogenicity and antifungal resistance, *Candida* species have gained considerable attention as important pathogenic organisms^3^. 

Secretory proteinases, as *Candida* virulence factors, can improve the potential of fungal organisms to colonize and penetrate into the host tissue and disrupt the immune system^4^. Phospholipase production is another major virulence factor of *C. albicans*, which binds the fungus to the target tissue and generates a pathway to enter the tissue following the hydrolysis of phospholipids and degradation of cell membranes^5^. In addition, haemolysin, as another extracellular enzyme, contributes to the invasion of yeasts through absorption of iron^6^. 

Biofilm formation, which is another virulence factor of *C. albicans,* plays a pivotal role in the pathogenesis of fungi through the mass production of pseudohyphae^7^ ^-^ ^9^. Biofilm contains dense yeast cells and hyphae, which can lead to antifungal resistance, as well as frequent recurrence of *Candida* infections. Studies indicate that *C. albicans* uses the nail as the substrate for biofilm formation. In fact, the production of biofilms in the nail acts as a source of continuous infection and leads to limited penetration of antifungal agents into the *Candida* biofilm^3^ ^,^ ^10^. Furthermore, long-term treatment and excessive exposure to antifungals lead to variable levels of resistance in *Candida* species. The purpose of this study was to determine the antifungal susceptibility profile of four antifungals *in vitro*, examine the production of aspartyl protease, phospholipase, haemolysin, and biofilm, and assess the relationship between these characteristics and the minimum inhibitory concentration (MIC) of antifungals for *C. albicans* isolates from patients with clinical onychomycosis in Iran.

## METHODS

### Fungal strains

This study was conducted on *C. albicans* isolates (n = 70), obtained from patients with onychomycosis, who were admitted to the Dermatology Clinic of Razi Skin Hospital in Tehran, Iran from November 2017 to October 2018. The physiological characteristics of the isolates were identified using the germ tube test^11^ and colony color analysis on CHOROM agar *Candida* medium (CHOROMagar Candida, France)^12^. All isolates were *stored*at*−*20°*C until*use. Before the experiments, these isolates *were grown*on Sabouraud dextrose agar (SDA, Acumedia, Baltimore, Maryland, USA) supplemented with 100 μg/ml cycloheximide (Sigma, Steinheim,Germany) and incubated at a temperature ranging from 25-30°C.

 Next, genomic DNA was extracted using glass beads and phenol:chloroform:isoamyl alcohol (25:24:1) method^13^. All clinical *C. albicans* isolates were confirmed through PCR amplification of the hyphal wall protein 1 (*HWP1*) gene, using forward (5′GCTACCACTTCAGAATCATCATC-3′) and reverse (5′ GCACCTTCAGTCGTAGAGACG-3′) primer pairs^14^. 

### Antifungal susceptibility test

The antifungal susceptibility profiles were determined *in vitro* using the broth microdilution method, according to the M27-A3 guidelines of the Clinical and Laboratory Standards Institute (CLSI)^15^. All isolates were evaluated against amphotericin B (AMB), fluconazole (FLC), itraconazole (ITC), and voriconazole (VRC) (Sigma-Aldrich Chemical Corporation, St. Louis, MO, USA). Briefly, each well of the 96-well round-bottom microtiter plate consisted of 100 μL of RPMI-1640 medium, 64-0.06 μg/mL of FLC, and 16-0.016 μg/mL of AMB, ITC, and VRC with 1-5×10^3^ cells/mL of yeast. The plates were then incubated at 35°C for 48 h. *Candida parapsilosis* ATCC 22019 and *Candida krusei* ATCC 6258 were also used as quality controls. 

### Production of hydrolytic enzymes

The activity of aspartyl protease was determined using bovine serum albumin (BSA) (pH, 5.0) agar assay, as previously described^16^. The assay was performed by adding 10 µL of cell suspension (McFarland turbidity, 0.5) to a sterile paper disk, which was placed on the surface of BSA medium, followed by incubation at 37°C for six days. Next, the plates were fixed with 20% trichloroacetic acid (TCA) and were stained with 1.25% Amido Black dye^17^. *C. albicans* ATCC 10231 was used as the positive control. 

The production of phospholipases by *C. albicans* isolates was achieved using egg yolk agar plate method^18^. The test was conducted by adding 10 μL of cell suspension (0.5 McFarland turbidity), inoculated onto SDA plates (containing 1 M NaCl, 0.005 M CaCl_2_, and 8% sterile egg yolk emulsion), followed by incubation at 37°C for four to five days. *C. albicans* SC 5314 strain was used as the positive control. In addition, the hemolytic activity of *C. albicans* isolates was evaluated in the SDA medium with 7% blood and 3% glucose^19^. Similarly, 10 µL of the yeast suspension (10^8^ cells) was inoculated onto the surface of the medium, and each plate was incubated at 37°C for two to three days. *C. albicans* ATCC 10261 was used as the control strain.

Furthermore, enzyme activity (P_rz_, P_z_, and H_z_) was determined for each isolate by measuring the ratio of colony diameter to the colony diameter plus the area of zone. According to the results, four classes of isolates were identified with respect to the enzyme activity zone: 1, negative activity; 0.999-0.700, weak producer; 0.699-0.400, moderate producer; and < 0.399, strong producer^18^. Each isolate was tested in duplicate.

### Biofilm formation

Biomass was assessed using crystal violet assay^20^. Briefly, *C. albicans* isolates, which were grown on SDA at 37°C for 24 h, were washed, and suspension was adjusted to 3×10^7^ CFU/mL. Next, 20 µL of the yeast cell suspension and 180 µL of Sabouraud dextrose broth (SDB) supplemented with 60 g of glucose, were inoculated into flat-bottom 96-well microplates. The plates were then incubated at 35°C for 24 h. After incubation and washing, the plate walls were stained with 1% crystal violet for 15 min, following which 200 μL of 95% ethanol was added to each well^21^. 

The optical density (OD) was measured using an ELISA reader at 590 nm. The OD cut-off point (ODc) for biofilm formation was determined as three standard deviations above the mean absorbance of the negative control^22^. The isolates were classified into four groups according to OD_590_: non-biofilm producer, OD_590_ ≤ ODc; weak producer, ODc < OD_590_ ≤ 2 × ODc; moderate producer, 2 × ODc < OD_590_ ≤ 4 × ODc; and strong producer, OD_590_ nm > 4 × ODc (1, 2). The ODc measured in this study was 0.365.

### Scanning electron microscopic (SEM) study of *Candida* biofilms 

In this study, *C. albicans* isolates with the highest biofilm-forming ability among the ones tested were selected and analyzed through scanning electron microscopy (SEM), according to a previously described method^23^. After incubation and removal of free cells by washing, biofilms and sticky cells were fixed with glutaraldehyde and incubated at 4°C overnight. After the biofilms were washed with PBS, they were dehydrated with various dilutions of ethanol (50%, 70%, 80%, 95%, and 100%), and the plates were dried in air. Finally, the biofilms were coated with gold and studied through SEM.

### Statistical analysis

Statistical analysis was performed using SPSS version 22.0 (SPSS Inc., Chicago, IL, USA). Normality assumption was evaluated using the Kolmogorov-Smirnov test, which showed that the data were not normally distributed. Moreover, Spearman's rank-order correlation test was used to evaluate the correlation between antifungal MICs, extracellular enzymes, and biofilm production. The median values of variables and 95% confidence interval for median (95% CI) were compared for each group of antifungal profile, using Mann-Whitney U test. P-value < 0.05 was considered statistically significant.

## RESULTS

### Identification and antifungal susceptibility profile of *C. albicans* isolates

The results of germ tube test in the serum and green colony formation on CHOROM agar candida medium were confirmed using PCR-*HWP1* assay for *C. albicans* isolates (~1000 bp). The antifungal susceptibility test was performed *in vitro* based on the CLSI M27-A3 guidelines, shown in Table 1. All *C. albicans* isolates (100%) were susceptible to AMB and VRC at concentrations ≤ 1 μg/mL, whereas eight isolates (11.4%) were resistant to FLC (MIC ≥ 64 μg/mL), and 19 isolates (27.1%) were susceptible-dose dependent (SDD). Furthermore, six isolates (8.6%) showed the highest MIC to ITC (MIC ≥ 1 μg/mL), while 18 isolates (25.7%) were SDD. 

**TABLE 1:** *In vitro* antifungal susceptibility testing of 70 *Candida albicans* isolated from patients with onychomycosis.

Antifungal	Range	MIC(µg/ml)	GM	CLSI		
		MIC50/MIC90		S	SDD	R
**AMB** ^1^	0.016-0.125	0.062/0.125	0.054	70 (100%)	-	-
**FLC** ^2^	0.125-64	2**/**64	2.48	43 (61.4%)	19 (27.1%)	8 (11.4%)
**ITC** ^3^	0.031-4	0.125**/**0.5	0.129	46 (65.7%)	18 (25.7%)	6 (8.6%)
**VRC** ^4^	0.016-0.125	0.062**/**0.125	0.063	70 (100%)	-	-

**MIC:** minimal inhibitory concentration; **CLSI:** Clinical and Laboratory Standards Institute; **GM:** geometric mean; **S:** susceptible; **SDD:** susceptible-dose dependent; **R:** resistant. ^1^Amphotericin B (AMB); ^2^Fluconazole (FLC); ^3^Itraconazole (ITC); ^4^Voriconazole (VRC).

### Production of hydrolytic enzymes and biofilm formation

**FIGURE 1:**
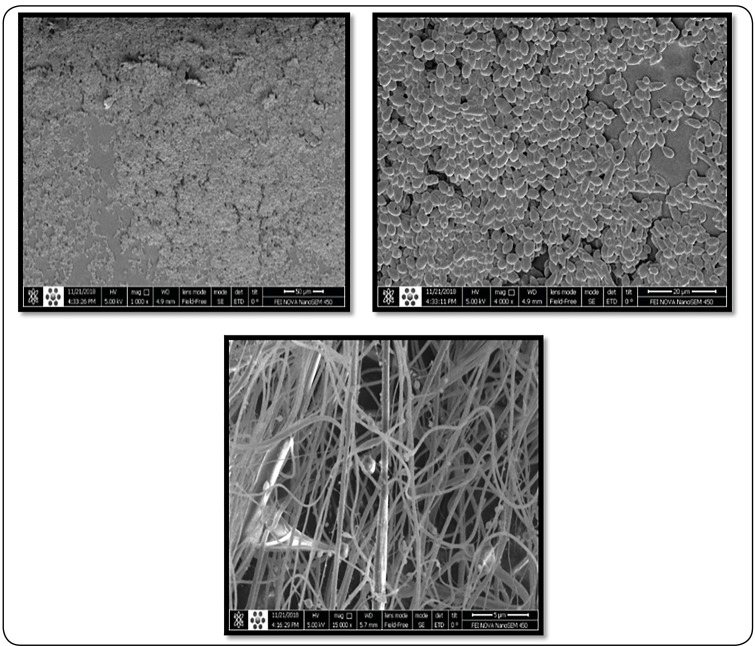
Biofilm structure of the network of dense yeast cells and hyphae for *C. albicans* with strong biofilm production (OD_590_ > 1.46). Scanning electron microscopy images acquired after biofilm growth in ­SDB after 24 h with 1000X, 4000X, and 15000 X, magnification.

Evaluation of proteinase production showed that 72.85% of *C. albicans* isolates produced aspartyl protease (P_rz_ range, 0.32 to 0.69). In addition, phospholipase activity was detected in 61.42% of *C. albicans* isolates (P_z_ range, 0.22 to 0.96). Overall, 17 isolates (24.3%) were considered as strong producers (P_z_ range, 0.22 to 0.39), 15 isolates (21.4%) were classified as moderate producers (P_z_ range, 0.43 to 0.69), and 11 isolates (15.7%) were considered as weak producers (P_z_ range, 0.73 to 0.96). In this study, we observed that 94.28% of *C. albicans* isolates from the nail bed of patients with onychomycosis were able to induce hemolytic activity (H_z_ range, 0.33 to 0.69). In total, 22.9% of isolates were considered as strong producers (H_z_ range, 0.33 to 0.38), while 71.4% of isolates were classified as moderate producers (H_z_ range, 0.47 to 0.69). Additionally, biofilm production of 70 *C. albicans* isolates were classified into four groups according to OD_590_: non-biofilm producers (n = 47; 67.14%), weak (n = 7; 10%), moderate (n = 13; 18.6%), and strong (n = 3; 4.3%). Furthermore, Figure 1 shows the SEM results of the network of dense yeast cells and hyphae by *C. albicans* with strong biofilm producer (OD_590_ > 1.46). 

The results also showed significant correlations between phospholipase activity and biofilm production in *C. albicans* isolates (P = 0.004, r_s_ = 0.339). In addition, there was no significant relationship between aspartyl protease, hemolytic activities, and biofilm production.

### Correlation between antifungal susceptibility profile and virulence factors

**FIGURE 2:**
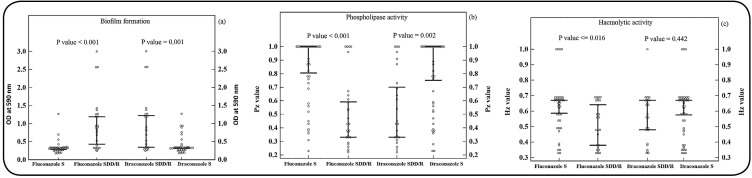
Differences in the median (95% CI for median) phospholipase index Pz values, hemolytic activity Hz values, and optical density at 590 nm of formed biofilms between strains with different fluconazole and itraconazole susceptibility profiles with Mann-Whitney test. **(a)** Absorbance value (OD 590 nm) of formed biofilms with FLC and ITC susceptibility profiles. **(b)** Median P_z_ values of phospholipase of strains with FLC and ITC susceptibility profiles. **(c)** Median H_z_ values of hemolytic activity of strains with FLC and ITC susceptibility profiles. In all the cases, the differences between the medians of groups with different susceptibility profiles were statistically significant (P < 0.05), except for H_z_ values of hemolytic activity with itraconazole susceptibility profile (P>0.05). **S:** susceptible; **SDD:** susceptible-dose dependent; **R:** resistant; 95% **CI:** 95% confidence interval.

Evaluation of the correlation between antifungal MICs and virulence factors revealed significant relationships between FLC MIC and biofilm formation (P = 0.05, r_s_ = 0.725), ITC MIC and biofilm formation (P = 0.002, r_s_ = 0.498), FLC MIC and phospholipase production (P = 0.05, r_s_ = 0.455), ITC MIC and phospholipase production (P = 0.003, r_s_ = 0.345), and FLC MIC and hemolytic production (P = 0.011, r_s_ = 0.302) (Table 2). The differences in the median production value of phospholipase, haemolysin, and biofilm formation by *C. albicans* isolates with their susceptibility profile against fluconazole and itraconazole antifungal are shown in Figure 2.

**TABLE 2:** Correlation between virulence factors and minimum inhibitory concentrations of two antifungals in 70 *Candida albicans* isolated from patients with onychomycosis.

Virulence factors (rs) and P value*				
Antifungal	Aspartyl protease	Phospholipase	Haemolysin	Biofilm
Fluconazole	0.054(0.658)	0.455(0.05)*	0.302(0.011)*	0.725(0.05)*
Itraconazole	0.306 (0.124)	0.345(0.003)*	0.065**(0.594)**	0.498(0.002)*

*P < 0.05 (**in bold**) were considered statistically significant.

r_s_: Spearman’s correlation coefficient.

## DISCUSSION

*Candida* species are regarded as the second most common cause of onychomycosis after dermatophytes. However, the growing prevalence of onychomycosis due to different varieties of *Candida* species has been reported in several parts of the world in recent years^2^. The increasing resistance of *C. albicans* to antifungals possibly indicates the relationship between the antifungal resistance mechanisms and production of biofilms and extracellular enzymes^3^ ^,^ ^10^.

The antifungal susceptibility pattern identified in this study indicated that all isolates were highly susceptible to AMB (100%) and VRC (100%). According to the present results, which are supported by previous studies, susceptibility to AMB and VRC was detected in *C. albicans* isolates^24^ ^,^ ^25^. In contrast, Pakshir et al. reported that 43% of *C. albicans* isolates from patients with onychomycosis were resistant to VRC in Iran^26^. In addition, our results showed that 11.4% and 8.6% of *C. albicans* isolates were resistant to FLC and ITC, respectively. These findings are consistent with those of previous studies conducted in Portugal and China^23^ ^,^ ^24^. 

Moreover, Khosravi et al. reported that 85.7% of *Candida* species isolated from the nails were susceptible to FLC in Iran^27^. The cause of the difference in antifungal resistance patterns of *Candida* species is related to clinical samples, immune system status, extensive use of azoles, and different distribution patterns of isolates in different geographical areas.

The extracellular enzymes of *C. albicans* play a pivotal role in binding, invasion, and destruction of the host cellular structure. *C. albicans* has the ability to synthesize aspartyl proteinase through the destruction of elastin, which is involved in the invasion of host tissues. Based on our results, the highest proteinase activity was observed in 72.85% of *C. albicans*. Previous studies have reported the rate of proteinase production to be 40%^28^, 82.1%^29^, and 97%^30^. Additionally, phospholipases are responsible for the hydrolysis of phospholipids, which help *Candida* invade the cells and destroy the host cell membrane. 

According to our results, 61.42% of *C. albicans* isolates produced phospholipases. In a study conducted by Sav et al., the highest phospholipase activity was reported in *C. albicans* isolates from nail samples^25^. Ramos Lde et al. reported that all *C. albicans* isolates (100%) from cutaneous candidiasis produced both aspartyl protease and phospholipase^31^. In addition, El-Houssaini et al. reported that 42% of *C. albicans* isolates from vaginal swab specimens produced phospholipase^28^. 

Iron uptake through haemolysin is an essential requirement for the growth and invasion of fungi^5^. In this study, 66 *C. albicans* isolates (94.28%) produced haemolysin, which is in agreement with the findings of recent studies^6^ ^,^ ^29^ ^,^ ^32^, suggesting that hemolytic activity is an important virulence factor of *C. albicans*. Moreover, biofilm production, as another virulence factor, plays a major role in the pathogenesis of *Candida* species^3^. Biofilm formation on the nail bed shows that *C. albicans* uses the nail as a substrate for producing thick biomasses of biofilm^10^.

In the present study, 23 isolates (32.85%) from 70 *C. albicans* isolates from patients with onychomycosis showed the ability to form biofilms at weak, moderate, and strong levels *in vitro*. Other researchers have reported the ability to form biofilms in 42.9%^33^, 40.3%^34^, and 33.3%^21^ of *C. albicans* and indicated the relationship between high biofilm production and pathogenesis. Furthermore, multiple studies have shown that the rate of proteinase, phospholipase, haemolysin, and biofilm production is variable, depending on the location of infection and number of *Candida* isolates. Analysis of the effect of extracellular enzyme activity on biofilm production showed a significant correlation between phospholipase activity and biofilm production in *C. albicans* isolates (P< 0.05). 

Similar results have been reported by El-Houssaini et al. from Egypt^28^ and Deorukhkar et al. from India^35^. They revealed a significant positive correlation between biofilm formation and phospholipase production. Some of the proposed hypotheses in this area suggest that the importance of biofilm formation by *Candida* species is related to the reduction of azole drug penetration into the biofilm matrix, reduced metabolism of biofilm cells, and increased expression of gene resistance, associated with antifungal resistance^36^ ^,^ ^37^. 

The present results showed a significant positive correlation of FLC/ITC MICs with biofilm formation and phospholipase production in *C. albicans in vitro*. Furthermore, there was a significant association between FLC MIC and haemolysin production. Therefore, biofilm formation can play a significant role in the increased azole resistance of *C. albicans* isolates from clinical specimens. 

The current study confirmed the effect of extracellular enzymes on biofilm production and determined the relationship between virulence factors and antifungal resistance in *C. albicans* isolates from patients with clinical onychomycosis *in vitro*. These results can contribute to a better understanding of biofilm formation and extracellular enzymes as potential targets against antifungal resistance. Further studies on genetic and molecular levels are required to better understand the association between virulence factors and antifungal resistance of *C. albicans* clinical strains. Moreover, the relationship between virulence factors and antifungal resistance may highlight new therapeutic strategies, based on the involvement of the virulence mechanism in the effectiveness of treatment.

 The code of ethics for this study is **IR.QUMS.REC.1396.283**.
